# Association Between Depression and Anxiety Status With Uptake of Colorectal Cancer Screening Among US Adults: A Population-Level Study

**DOI:** 10.7759/cureus.42659

**Published:** 2023-07-29

**Authors:** Radhey Patel, Prince C Akahara, Mohammed Raaid O Musa, Obiamaka P Okereke, Chander Puri, Saare Abera, Obiaku U Okoronkwo, Joy Iroro, Abigail O Dan-Eleberi, Okelue E Okobi, Ogechukwu Nwachukwu

**Affiliations:** 1 Psychiatry and Behavioral Sciences, Avalon University School of Medicine, Willemstad, CUW; 2 Mental Health, Manor Clinic, Edmonton, CAN; 3 Internal Medicine, University of Ilorin Teaching Hospital, Ilorin, NGA; 4 Family Medicine, Ebonyi State University, Ebonyi, NGA; 5 Medical School, Gian Sagar Medical College and Hospital, Patiala, IND; 6 Internal Medicine, American University of Integrated Sciences, St. Michael, BRB; 7 School of Medicine, Kwame Nkrumah University of Science and Technology, Kumasi, GHA; 8 Internal Medicine, All Saints University School of Medicine, Dominica, DMA; 9 Internal Medicine, University of Ghana Medical School, Accra, GHA; 10 Family Medicine, Medficient Health Systems, Laurel, USA; 11 Family Medicine, Lakeside Medical Center, Belle Glade, USA; 12 Internal Medicine, St. Helens and Knowsley Teaching Hospitals NHS Trust, Merseyside, GBR

**Keywords:** mental health disorders, colo rectal cancer, anxiety, depression, screening

## Abstract

Background: Individuals with different mental disorders tend to experience higher rates of colorectal cancer (CRC)-related mortality compared to the general population. Discrepancies in CRC screening behaviors have been suggested as a potential contributing factor to this difference in mortality. However, existing evidence on this topic has been inconclusive and conflicting.

Objective: This study aims to explore the relationship between mental health status (specifically, depression and/or anxiety) and the uptake of CRC screening. To achieve this, a larger and nationally representative sample from the adult population of the United States was utilized.

Methods: We employed a cross-sectional approach using data from the 2019-2020 edition of the Health Information National Trends Survey (HINTS). The study examined disparities in CRC screening between individuals with self-reported history of depression diagnosis and the general population. Chi-square tests were used for analysis. Multivariable logistic regression models were applied to adjust for variables such as gender, age, education level, race, comorbidities, healthcare access, smoking status, household income, geographical residence, and insurance status. Adjusted odds ratios (AORs) with 95% confidence intervals (CIs) were reported.

Results: The findings of the study indicated that out of 5,398 eligible individuals, approximately 1,220 (weighted percentage: 22.8%) reported experiencing depression and/or anxiety, and approximately 4,154 (weighted percentage: 68.9%) reported adherence to colorectal cancer screening. In the bivariate analysis, there was no significant difference in participation in colorectal cancer screening between individuals with and without depression and/or anxiety (72.0% vs. 68.0%). Similarly, after adjusting for sociodemographic and health-related factors, the study found that the odds of participating in colorectal cancer screening did not vary based on an individual's depression status (OR 1.34, 95% CI 0.94-1.91, P = 0.05).

Conclusion: Individuals with depression participate in colorectal cancer screening at comparable rates to the general population. The findings of this study suggest that factors beyond CRC screening may play significant roles in the higher CRC-associated mortality rate. Therefore, further research is needed to uncover the various mechanisms contributing to the increased cancer-related mortality rates among susceptible populations.

## Introduction

Individuals with mental disorders have been found to have higher rates of illness and death compared to the general population [1]. Extensive research has consistently shown that those with mental health disorders tend to die 10 to 20 years earlier than individuals without such disorders [1,2]. Furthermore, there is growing concern that the mortality gap has been widening over time [3].

Among the various causes of premature mortality, cancer stands out as a significant contributor, particularly among individuals with mental disorders [4,5]. Colorectal cancer (CRC) is one of the most prevalent types of cancer globally, accounting for over 693,900 deaths in 2012 [6]. In the United States, CRC remains the second leading cause of cancer-related deaths among adults [7].

While the literature suggests that individuals with mental conditions may have lower cancer incidence compared to the general population, studies have consistently shown higher cancer mortality rates in this group [4]. Numerous research studies have sought to uncover the underlying reasons for these disparities in mortality rates. Some researchers have proposed that the differences may be due to delayed diagnosis and limited access to healthcare and treatment among individuals with mental disorders [4,8]. Another crucial factor contributing to the disparity is the lower utilization of preventive services, including early screening practices.

For CRC, the Centers for Disease Control and Prevention (CDC) in the United States recommends prompt detection through screening for individuals between the ages of 45 and 75 [9]. Timely detection of CRC through screening is essential for improving treatment outcomes. Screening allows for the identification of precancerous polyps or early-stage cancers, enabling timely intervention and potentially preventing disease progression. Nevertheless, prior research indicates that individuals with various mental conditions have not been receiving adequate rates of preventive screening for CRC. For instance, a study conducted by Molly et al. on 855 veterans revealed that patients with a history of one or more mental health diagnoses had higher CRC screening rates (57% vs. 47%, P < 0.01) according to bivariate analyses. Similarly, Mo et al. examined 591 individuals from community mental health services and found a comparable suboptimal pattern. Additionally, Xiong's analysis of 387 individuals showed that among eligible men and women over the age of 50, 38 out of 68 (56%) had never undergone screening for colorectal cancer. These findings underscore the need to address the issue of low preventive screening rates for CRC in individuals with mental health conditions [10-12]. Additionally, the impact of mental health disorders on CRC screening participation remains unclear. Existing research on the link between mental disorders and CRC screening has produced mixed and inconclusive results. While some studies suggest that individuals with mental disorders have lower rates of CRC screening compared to the general population, others have found no significant correlation between mental health status and screening for CRC [10,13-15]. This inconsistency emphasizes the need for further research to clarify the relationship and identify contributing factors.

Given that individuals with mental disorders, such as depression and anxiety, experience higher rates of CRC-related mortality, understanding the nuanced correlation between mental health status and CRC screening is crucial for identifying potential disparities in screening rates and developing targeted interventions to address the elevated CRC-related mortality in this vulnerable group. Therefore, the aim of this study is to evaluate the existing correlation between depression status and colorectal cancer screening, aiming to fill the existing gaps in the literature. We hypothesize that individuals with depression are more likely to have lower rates of CRC screening compared to those without depression.

## Materials and methods

Study design and study samples

We utilized the Strengthening the Reporting of Observational Studies in Epidemiology (STROBE) guidelines [16] to guide their study design. We conducted a cross-sectional research study using data from the Health Information National Trends Survey (HINTS). HINTS is a nationally representative household interview study that includes American adults aged 18 years and above who are not institutionalized. The National Cancer Institute (NCI) has been administering the HINTS survey since 2003, collecting information on health communication, behaviors, information technology, and cancer-related topics such as diagnosis, prevention, survivorship, and treatment. For this particular study, data were drawn from the HINTS 5th edition, specifically Cycles 3 and 4. Cycle 3 data were collected between January and May 2019, while Cycle 4 data were collected between February and June 2020.

Details about the data collection methods, including weighting and sampling processes, can be found in previous publications [17,18]. In brief, the HINTS 5 iterations employed a two-stage, stratified random sampling methodology. The first stage involved selecting non-vacant residential homes obtained from the Marketing Systems Group (MSG). The second stage involved selecting one adult from each household using the "Next Birthday" method. The residential homes database was categorized into two strata: "high-minority" (with over 34% Hispanic and African American populations) and "low-minority" (with below 34% Hispanic and African American populations). This stratification aimed to improve the accuracy of estimates for minority subpopulations. The survey participants were weighted to reflect the selection probabilities and ensure national representativeness in terms of age, marital status, gender, census region, race/ethnicity, and educational attainment. Each adult participant was assigned a full-sample weight and a set of 50 replicate weights. The replicate weights were used to compute the standard error of estimates using a jackknife replication technique known as delete one jackknife (JK1) [19].

Ethical approval

The HINTS 5 Cycles 3 and 4 were approved by the Westat Institutional Review Board and considered exempt from the US National Institutes of Health Office of Human Subjects Research Protections review due to the de-identification of the data. Since this study was a secondary data analysis from the HINTS data, ethical approval for this study was exempt. Under the Common Rule, analyzing publicly available data using a secondary data analysis technique does not require IRB approval as per ethical research guidelines. Generally, publicly available data that have already been de-identified, meaning they lack identifiable information about individuals, are exempt from IRB oversight. De-identified data refers to information that has been stripped of identifiers like names, addresses, social security numbers, or any other details that could potentially identify individuals.

Study design and participants

A cross-sectional design was employed to evaluate participant responses from both HINTS 5 Cycle 3 (H5C3) and HINTS 5 Cycle 4 (H5C4). The combined datasets from H5C3 and H5C4 included responses from 9,303 respondents, with 5,438 respondents from Cycle 3 and 3,865 respondents from Cycle 4. The overall response rates for households were 30.3% for H5C3 and 37% for H5C4, which were comparable to previous HINTS iterations. It's important to note that participants surveyed in each cycle were different individuals, and therefore, this was not a repeated measures design. For the purpose of this study, participants were categorized into two groups: the depression/anxiety group and the control group (non-depressed and non-anxious individuals). The grouping was based on participants' responses to a survey item regarding depression diagnosis, as described in the measures section. Consistent with previous studies, participants who responded "yes" were classified as having depression/anxiety [20,21].

In this study, the inclusion criteria were based on the eligibility age for colorectal cancer screening [9]. Participants below 45 years and above 75 years of age were excluded. The final sample consisted of 5,398 individuals who provided responses to survey questions about depression status and colorectal cancer screening.

Measures

The main objective of this study was to assess the relationship between mental health status and participation in colorectal cancer screening among eligible adults in the United States. To achieve this objective, information from the survey items in both H5C3 and H5C4 was used as follows:

Outcome 

The primary outcome of interest to the researchers was screening for CRC. This was determined by analyzing participants' responses to a research question in a yes or no format. Participants were asked if they had undergone any of the screening tests for colon cancer, such as sigmoidoscopy, colonoscopy, and stool blood test.

Exposure

Participants' responses to various questions were used to determine participants with depression. They were asked if they had ever been diagnosed with depression or any anxiety disorder by a physician or healthcare professional ("Has a doctor or other health professional ever told you that you had depression or anxiety disorder?"). The response options were yes or no.

Covariates

The study included several participant attributes, such as gender, age, ethnicity/race, educational level, comorbidities, household income, insurance status, access to healthcare provider, smoking status, and geographical residence. Age was categorized into two groups: 45-60 years and 65-74 years. Ethnicity/race was classified as Hispanic, non-Hispanic black, non-Hispanic white, and others. Educational level was divided into three categories: high school and below, some college, and college graduate/postgraduate. Annual household income was categorized as below $20,000, $20,000-$34,999, $35,000-$49,999, $50,000-$74,999, and above $75,000. Participants were considered to have comorbidities if they reported having one or more of the following conditions: hypertension, diabetes mellitus, lung disease, or heart disease. Rural and urban residences were defined using Rural-Urban Continuum (RUC) Codes, with codes 1-3 representing urban residences and codes 4-9 representing rural residences. Smoking status was categorized as never smokers, current smokers, and former smokers.

Statistical analysis

Descriptive statistics were used to describe the study sample. Weighted percentages were presented, and Chi-squared tests were used to assess correlations between socio-demographic attributes, depression status, and colorectal cancer screening uptake. Multivariable logistic regression models were employed to adjust for age, race/ethnicity, gender, educational level, comorbidities, household income, access to healthcare provider, smoking status, geographical residence, and insurance status. The models aimed to evaluate the effects of depression status on colorectal cancer screening. Adjusted odds ratios (AORs) with 95% confidence intervals were calculated and reported. All statistical analyses were conducted using Stata version 17.0 (StataCorp., College Station, TX, USA) with the "svy" command. The jackknife replicate weights and final person weights provided in the H5C3 and H5C4 datasets were utilized to estimate national-level values and corresponding standard errors. A statistical significance level of 0.05 was set (p < .05).

## Results

The study sample consisted of 5,398 individuals, including 1,220 (weighted percentage of 22.8%) who self-reported having depression, and approximately 4,154 (weighted percentage of 68.9%) who reported participating in colorectal cancer screening. Overall, around 63.5% of the participants were aged between 45 and 60 years. Females accounted for 50.4% of the sample, and non-Hispanic whites made up 67.8% of the participants. Furthermore, 27.6% of the participants reported having a college/postgraduate education, while 71.0% had access to healthcare services, and nearly 14.3% were active smokers.

The study findings revealed that women had a higher prevalence of self-reported depression, with 27.0% compared to men at 18.5%. Similar patterns were observed among younger individuals aged 45-60 years (25.1%) compared to older individuals aged 61 to 74 years (18.9%). Moreover, individuals with a high school education or below were more likely to self-report depression at 24.8% compared to those with a college education at 18.6%. Similar trends were observed in individuals with lower incomes, particularly households earning less than $20,000 annually (39.7%), compared to those from households earning over $75,000 annually. Additionally, individuals with access to regular healthcare providers had a higher prevalence of self-reported depression at 25.9% compared to those without regular healthcare access at 15.6%. The demographic distribution of the entire population based on depression status is shown in Table 1 below.

**Table 1:** Sociodemographic characteristics by depression and/or anxiety status among sampled population (-) Intentionally left blank

Demographic variables	Total (n= 5398), %	Non-depressed/Non anxious (N = 4,178), %	Depression/Anxiety (N = 1,220), %	Test-Statistic	p-value
Gender	-	-	-	16.1	<0.001
Female	50.4	73.0	27.0	-	-
Male	49.6	81.5	18.5	-	-
Age Group (year)	-	-	-	10.7	0.002
45 – 60	63.5	74.9	25.1	-	-
61 – 75	36.5	81.1	18.9	-	-
Education	-	-	-	3.9	0.024
High school or less	31.7	75.2	24.8	-	-
Some college	40.7	75.7	24.3	-	-
College graduate or more	27.6	81.4	18.6	-	-
Household Income	-	-	-	16.4	<0.001
Less than $20,000	16.2	60.3	39.7	-	-
$20,000 - $34,999	10.7	72.1	27.9	-	-
$35,000 - $49,999	12.8	78.6	21.4	-	-
$50,000 - $74,999	18.0	79.7	20.3	-	-
$75,000 or more	42.3	83.4	16.6	-	-
Race	-	-	-	2.73	0.048
White	67.8	75.5	24.5	-	-
Black/African American	11.3	80.6	19.4	-	-
Hispanic	13.8	80.9	19.1	-	-
Others	7.1	81.7	18.3	-	-
Insurance status	-	-	-	0.07	0.798
No	5.9	76.0	24.0	-	-
Yes	94.1	77.1	22.9	-	-
Residence	-	-	-	0.16	0.687
Urban	86.8	77.0	23.0	-	-
Rural	13.2	78.1	21.9	-	-
Comorbidity	-	-	-	22.2	<0.001
None	41.8	82.7	17.3	-	-
At least one	58.2	73.1	26.9	-	-
Regular Provider	-	-	-	17.4	<0.001
No	29.0	84.4	15.6	-	-
Yes	71.0	74.1	25.9	-	-
Smoking status	-	-	-	15.9	<0.001
Never	58.2	80.4	19.6	-	-
Former	27.5	77.3	22.7	-	-
Current	14.3	64.2	35.8	-	-

In the bivariate analysis presented in Table 2 below, it was observed that individuals who self-reported undergoing colorectal cancer screening were more likely to be in older age groups (88.0% were aged 61 to 74 years) compared to younger age groups (58.0% were aged 45 to 60 years). Moreover, individuals with a college education (73.8%) were more likely to undergo screening than those with a high school education or below (63.5%). Additionally, individuals with insurance (71.3%) were more likely to undergo screening compared to those without insurance (28.2%), and those with access to a regular health provider (76.4%) were more likely to undergo screening than those without a regular provider (50.3%). However, there was no significant variation in colorectal cancer screening based on individual depression status (72.0% vs. 68.0%). Among different racial groups, Hispanics had the lowest prevalence of colorectal cancer screening. Table 2 below provides the demographic distribution of the population based on colorectal cancer screening status.

**Table 2:** Sociodemographic characteristics by colorectal cancer screening status among sampled population (-) Intentionally left blank

Demographic variables	Total (n=5398), %	No, Screening (n=1244), %	Yes, screening (n=4154), %	Test-Statistic	p-value
Gender	-	-	-	0.06	0.802
Female	50.4	30.7	69.3	-	-
Male	49.6	30.1	69.9	-	-
Age Group	-	-	-	310.0	<0.001
45yr – 60yr	63.5	42.0	58.0	-	-
61yr – 75yr	36.5	12.0	88.0	-	-
Education	-	-	-	9.10	<0.001
High school or less	31.7	36.5	63.5	-	-
Some college	40.7	29.4	70.6	-	-
College graduate or more	27.6	26.2	73.8	-	-
Household Income	-	-	-	2.37	0.053
Less than $20,000	16.2	34.3	65.7	-	-
$20,000 - $34,999	10.7	36.6	63.4	-	-
$35,000 - $49,999	12.8	31.5	68.5	-	-
$50,000 - $74,999	18.0	32.2	67.8	-	-
$75,000 or more	42.3	27.6	72.4	-	-
Race	-	-	-	18.0	<0.001
White	67.8	27.4	72.6	-	-
Black/African American	11.3	26.4	73.6	-	-
Hispanic	13.8	49.4	50.6	-	-
Others	7.1	42.3	57.7	-	-
Insurance status	-	-	-	137.6	<0.001
No	5.9	71.8	28.2	-	-
Yes	94.1	28.7	71.3	-	-
Residence	-	-	-	0.84	0.363
Urban	86.8	30.7	69.3	-	-
Rural	13.2	33.6	66.4	-	-
Comorbidity				46.0	<0.001
None	41.8	39.6	60.4	-	-
At least one	58.2	25.3	74.7	-	-
Regular Provider	-	-	-	105.2	<0.001
No	29.0	49.7	50.3	-	-
Yes	71.0	23.6	76.4	-	-
Smoking status	-	-	-	15.9	<0.001
Never	58.2	31.0	69.0	-	-
Former	27.5	24.8	75.2	-	-
Current	14.3	42.4	57.6	-	-
Depression/Anxiety Status	-	-	-	2.08	0.153
No	77.2	32.0	68.0	-	-
Yes	22.8	28.0	72.0	-	-

After adjusting for health and sociodemographic factors, we found no significant variation in the odds of participating in colorectal cancer screening based on individual depression status (OR 1.34, 95% CI 0.94, 1.91; P = 1.05). Please refer to Table 3 below for detailed information.

**Table 3:** Multivariable logistic regression of the association between self-reported depression/anxiety status with uptake of colorectal cancer screening sample (-) Intentionally left blank

Demographic variables	Colorectal cancer screen Adjusted Odds Ratio, 95% CI	p-value
Depression/Anxiety Status	-	-
No (reference)	1.00	-
Yes	1.34 (0.94, 1.91)	0.105
Gender	-	-
Female (reference)	1.00	-
Male	1.04 (0.81, 1.33)	0.768
Age Group	-	-
45yr – 60yr (reference)	1.00	-
61yr – 75yr	5.04 (3.93, 6.47)	<0.001
Education	-	-
High school or less	1.00	-
Some college	1.47 (1.08, 2.02)	0.016
College graduate or more	1.51 (1.09, 2.08)	0.013
Household Income	-	-
Less than $20,000	1.00	-
$20,000 - $34,999	0.80 (0.48, 1.33)	0.386
$35,000 - $49,999	1.02 (0.61, 1.71)	0.937
$50,000 - $74,999	0.90 (0.60, 1.36)	0.609
$75,000 or more	1.23 (0.81, 1.86)	0.323
Race	-	-
White	1.00	-
Black/African American	1.90 (1.30, 2.80)	0.001
Hispanic	0.58 (0.42, 0.80)	0.001
Others	0.60 (0.34, 1.07)	0.084
Insurance status	-	-
No	1.00	-
Yes	4.40 (2.70, 7.30)	<0.001
Residence	-	-
Urban	1.00	-
Rural	0.74 (0.49, 1.13)	0.162
Comorbidity	-	-
None	1.00	-
At least one	1.51 (1.18, 1.94)	0.001
Regular Provider	-	-
No	1.00	-
Yes	2.20 (1.62, 2.97)	<0.001
Smoking status	-	-
Never	-	-
Former	0.93 (0.67, 1.28)	0.638
Current	0.63 (0.44, 0.89)	0.010

## Discussion

Individuals facing mental disorders may encounter unique obstacles in accessing medical care, including preventive services. Therefore, enhancing access to preventive services for individuals with mental disorders holds significant importance for public health. The objective of this study was to evaluate the existing correlations between mental health disorders and CRC screening. Specifically, we examined the differences in CRC screening participation between individuals with depression and/or anxiety and those without. The study utilized a nationally representative sample of 5,398 adults residing in the United States who had depression. The major findings of the study indicate that approximately 72% of eligible adults with depression participated in CRC screening. Importantly, contrary to the hypothesized relationship, the study revealed no significant variation in CRC screening based on depression status after adjusting for sociodemographic factors.

These findings align with earlier research on the correlation between mental disorders and CRC screening. For example, Hategekimana et al. conducted a study in Canada with 11,386 individuals aged 50 to 74 years and concluded that there was no significant correlation between self-reported mental disorders and CRC screening through fecal occult blood tests (FOBT) [22]. Similarly, Yee et al. conducted a study with 606 female veteran participants aged 50 to 65 years and found that females with mental health disorders were as likely as females without mental disorders to undergo CRC screening [23]. Furthermore, a study based on the California Health Interview Survey, which included 15,535 individuals aged 50 years and above, found no association between mental disorders and CRC screening [15].

Several possible explanations exist for the lack of support for the hypothesis that a mental disorder diagnosis increases the risk of non-participation in CRC screening. Firstly, individuals with mental disorders may have more frequent contact with healthcare providers for managing their mental health conditions. This frequent interaction may provide more opportunities for healthcare professionals to discuss and recommend preventive measures such as CRC screening. Additionally, the integration of primary care and mental health services has become increasingly common in healthcare settings [24-26]. In such models, healthcare providers collaborate to ensure that patients receive appropriate preventive care. This collaborative approach between mental health and primary care providers may contribute to improved CRC screening rates among individuals with mental disorders.

Despite the findings of this study, previous research has yielded divergent conclusions. Some studies have reported negative correlations between CRC screening uptake and mental health diagnoses [10,14,27]. The inconsistencies in findings may be attributed to various methodological factors. Firstly, there is substantial heterogeneity across studies due to differences in the types of mental health conditions studied and the composition of the sample population. While our study included participants with different anxiety and depression disorders in a nationally representative sample, other studies have focused on specific population groups such as veterans and women, and have examined a wider range of mental disorders including different psychotic disorders [10,14,27]. Secondly, differences in mental health measures (self-reported vs. standardized questionnaires) and the types of CRC screening utilized may also contribute to the divergent findings.

Although the study did not find substantial support for the presented hypothesis, it acknowledged other factors associated with CRC screening participation, such as being between the ages of 61 and 75, having some level of college or postgraduate education, having access to health insurance, and having access to healthcare providers. The study also identified reduced odds of CRC screening participation among active smokers and Hispanics. These findings highlight the need for targeted outreach and sustainable cancer prevention efforts to enhance CRC screening among vulnerable groups.

We considered certain study strengths and limitations while creating this manuscript. The utilization of HINTS provided a large dataset and sufficient power to detect differences. The use of survey weights allowed for generalization of the study results to the overall adult population in the United States. However, the study is limited by its cross-sectional nature, which restricts its ability to inform trends and patterns in CRC screening over time. Additionally, the cross-sectional design prevents causal conclusions from being drawn. The study's reliance on self-reported mental health diagnoses, both past and present, poses a limitation. Future studies should incorporate validated questionnaires and verify diagnoses using medical and clinical records. The survey design also made it challenging to assess other potentially relevant factors, such as the severity of mental disorders [28], specific types of mental disorders, and the use of psychotropic medications [29], which may confound the study outcomes. Furthermore, the data was limited to individuals residing in households and able to respond to survey questions, which limits the generalizability of the findings to individuals experiencing homelessness, who are at higher risk of mental disorders and have limited access to mental healthcare services [30-34]. Prospective studies should consider longitudinal datasets, employ objective measures, examine the experiences of individuals with mental conditions in psychiatric treatment settings, and explore the impact of knowledge on psychosocial factors affecting CRC screening participation, particularly within specific populations.

## Conclusions

In conclusion, contrary to the belief that people with depression have lower rates of CRC screening, our study found that individuals with depression participate in CRC screening at similar rates to the general population. This suggests that factors other than CRC screening may contribute to the higher CRC-related mortality observed among people with mental disorders. Further research is needed to identify and understand the mechanisms behind the elevated cancer-related mortality in this vulnerable population.
